# Progress Through Engagement: A Systematic Review and Thematic Synthesis of Qualitative Community‐Based Participatory Research in Vision Impairment

**DOI:** 10.1111/hex.70640

**Published:** 2026-03-31

**Authors:** Aikaterini Tavoulari, Jibraan Kidwai, Michael J. Proulx, Karin Petrini

**Affiliations:** ^1^ Department of Psychology University of Bath Bath UK; ^2^ The Centre for the Analysis of Motion, Entertainment Research and Applications (CAMERA) Bath UK; ^3^ Bath Institute for the Augmented Human (IAH) Bath UK

## Abstract

**Background:**

Vision impairment (VI) presents complex challenges that extend beyond clinical measures, affecting individuals' mental health, social participation and quality of life. Traditional research often overlooks these lived realities, prompting a growing interest in community‐based participatory research (CBPR) as a more inclusive and impactful approach.

**Methods:**

This systematic review examines the scope, frequency and methodological characteristics of CBPR studies involving people with VI, with a focus on qualitative research published between 1993 and 2024. Following PRISMA guidelines and PROSPERO registration, included studies were identified across diverse cultural contexts and participatory depths, from consultation to collaboration and user‐driven design. Using a dual‐stage thematic synthesis, we explored both the methodological approaches (‘how’) and the substantive content themes (‘what’) addressed in these studies. Participatory methods included photography and narrative storytelling, focus groups, creative workshops and final consultations, with many studies employing methodological triangulation. Although the search covered the period 1993–2024, all included studies were published between 2014 and 2024, reflecting the recent emergence of CBPR approaches in VI research.

**Results:**

Twelve studies met the inclusion criteria and were included in the final synthesis. Thematic findings revealed barriers to accessibility, cultural misalignment in interventions and the importance of user‐led adaptation strategies, showing that despite growing interest, CBPR remains underutilised in VI research.

**Conclusion:**

This review highlights its potential to bridge gaps between clinical innovation and lived experience, offering a roadmap for more equitable, context‐sensitive and user‐centred research practices.

**Patient or Public Contribution:**

Patients and members of the public were not directly involved in the design, conduct, or analysis of this systematic literature review. Instead, the study focus and synthesis were informed by documented experiences of people with vision impairment, derived from community‐based participatory studies, which contributed to the development of patient‐centred health material design.

## 1 Introduction

Vision impairment (VI) represents a significant global health challenge that affects individuals across all age groups and demographics [1]. While traditional research approaches such as epidemiological studies and clinical trials have contributed substantially to our understanding of VI prevalence, risk factors and treatment efficacy, they often fail to capture the nuanced lived experiences, daily challenges and priorities of those with a VI. This methodological limitation has led to research outcomes that, despite scientific validity, may lack relevance, accessibility, or practical applicability for the VI community.

The impact of VI extends beyond physical limitations, significantly affecting mental health, social participation and quality of life [2]. Individuals with VI are disproportionately affected by loneliness, isolation and depression relative to the general population [3], a disparity that may be further exacerbated by limited access to social opportunities such as exercise, which Richardson et al. [4] highlight as essential for social inclusion and well‐being. These challenges underscore the importance of developing research approaches that effectively capture and address the needs of this population while promoting their active involvement in finding solutions [5]. Including VI individuals not just as participants in research but as active co‐designers and co‐researchers is therefore critical to ensure that research addresses genuine needs, develops context‐appropriate solutions and ultimately produces outcomes with real‐world impact and user‐centred design [6]. Community‐based participatory research (CBPR) offers a collaborative approach that engages people with VI as partners in the research process, acknowledging their lived experiences and expertise [7].

CBPR exists on a spectrum of engagement, typically ranging from consultation (where community members provide input but researchers retain control), through involvement (where participants actively shape aspects of the research), to collaboration (where decision‐making is shared), and ultimately to empowerment or user‐driven research (where community members lead the research process) [8, 9, 10, 11, 12]. This approach marks a paradigm shift from traditional methodologies by emphasising equitable partnerships between researchers and community members [13, 14]. For instance, in a consultation‐level CBPR study, Theodorou and Meliones [5] engaged VI participants through interviews to inform the design of assistive navigation apps, ensuring user needs shaped key features and functionality. In contrast, Keene [15] conducted a collaboration‐level CBPR project where blind participants co‐constructed resources, analysed data and shaped recommendations to improve physical education, reflecting shared decision‐making and deeper engagement throughout the research process. These examples illustrate how CBPR can vary in depth of engagement, offering flexible yet meaningful routes to inclusive knowledge production.

This approach is particularly relevant in VI, where unique challenges distinguish it from other health conditions requiring specialised research considerations. Unlike many conditions with standardised experiences, VI presents with a highly individualised impact depending on onset age, progression rate, severity and specific visual function affected [16]. Clinical measures of visual acuity often fail to capture the functional impact on daily living, creating a disconnect between medical assessments and lived reality [17]. Moreover, while VI affects a minority of the global population overall [18], its prevalence increases sharply with age, with older adults carrying a disproportionately high burden of VI as highlighted in the WHO World Report on Vision and global disease burden analyses [19]. The challenges vary dramatically depending on whether vision loss occurred congenitally, during childhood development, or later in life, with particularly acute difficulties arising during transition phases such as entering education, workforce, or adjusting to progressive loss [20]. Notably, emerging research suggests that the timing of vision loss may also shape cognitive processing and neural adaptation, with congenital and early‐onset cases often associated with distinct patterns of brain reorganisation and sensory compensation compared to later‐onset cases [21].

Despite demonstrating scientific merit, several interventions targeting individuals with VI have failed to gain traction within affected communities. For instance, Pal et al. [22] evaluated a structured rehabilitation programme aimed at enhancing mobility and independence among adults with VI, yet uptake remained low due to limited cultural relevance and lack of user involvement in its design. Similarly, Metatla et al. [23] examined assistive technologies for young people with VI, uncovering barriers such as fragmented tools, inaccessible interfaces and misalignment with users' lived experiences. Tavoulari et al. [24] further demonstrated how the co‐design of digital tools with young people using a CBPR approach revealed challenges tied to cultural disparities and technological fragmentation; challenges that were often invisible to traditional research models. Complementing this, Hamideh Kerdar et al. [25] conducted a scoping review on digital accessibility, showing how the exclusion of users from design processes led to persistent failures in meeting basic accessibility standards.

These examples underscore the critical importance of integrating lived experience expertise into intervention development. Furthermore, communities across diverse cultural contexts have cultivated valuable adaptation strategies and informal support systems that remain siloed due to the absence of inclusive, collaborative research frameworks. CBPR offers a promising avenue to bridge this gap by engaging individuals with VI as co‐researchers. This approach enables the synthesis of varied lived experiences and innovations, fostering interventions that are not only clinically robust but also socially relevant, culturally sensitive and practically effective in promoting equity [26]. However, despite its potential, the prevalence and impact of CBPR within VI research remain underexplored and have not been systematically evaluated [27].

Accordingly, this systematic review pursues two primary objectives: first, to examine the scope and frequency of CBPR studies focused on VI, identifying the types of engagement, thematic focus and methodological approaches employed; and second, to explore how CBPR findings have been applied in practice to support individuals with VI and their communities. While some studies mention benefits and challenges, these were not consistently reported or analysed across the dataset and therefore do not form a core part of the results.

Given the methodological heterogeneity of CBPR studies in VI, our review adopted a thematic synthesis approach. This enabled systematic identification of methodological and experiential patterns across studies without engaging in the interpretive, theory‑generating processes typical of qualitative meta‑synthesis.

## 2 Methods

### 2.1 Protocol and Registration

This systematic review was pre‐registered with PROSPERO (Registration Number: CRD42024493802 Protocol). In addition, this review follows the Preferred Reporting Items for Systematic Reviews and Meta‐Analyses (PRISMA) guidelines [28], ensuring thorough documentation and reporting of our methodology and findings regarding CBPR in VI.

### 2.2 Search Strategy

We conducted comprehensive searches across multiple electronic databases, including Embase (containing PubMed, MEDLINE, Web of Sciences), Proquest (IBPS, IBSS, PTST), EBSCOhost (ERIC, CINAHL), ACM Digital Library, MIT Press Direct, Cochrane, ScienceDirect, APA PsycINFO, Scopus and specialised VI research databases [29]. Specifically, we searched two specialised VI databases: the Royal National Institute of Blind People (RNIB) research resources and the International Council for Education of People with Visual Impairment (ICEVI) database. Additional sources included thesis repositories (EthOS, Dart‐Europe, ProQuest Dissertations & Theses Global) and relevant organisational websites such as those of the Royal National Institute of Blind People (RNIB) and the International Council for Education of People with Visual Impairment (ICEVI).

The search string was designed for use in Advanced Search where possible. When Advanced Search was unavailable, the same string was used in a Basic Search:

(“vision impairment” OR “visual impairment” OR “visually impaired” OR “vision disorder” OR “vision disorders” OR “low vision” OR “partially sighted” OR “non sighted”) AND (“participatory research” OR “action research” OR “community‐based research”).

### 2.3 Eligibility Criteria

Inclusion Criteria:

1. We included studies published since 1993, a year that coincides with the rise of the internet and the expansion of digital research dissemination; thereby capturing a 30‐year span of key methodological shifts and innovations in disability research [30]. While the social model of disability has earlier origins in [31]' s work, the early 1990s saw the broader adoption of participatory approaches in disability studies, with seminal publications establishing frameworks for collaborative research [32]. Although the search strategy covered a 30‑year period (1993–2024), no eligible CBPR studies involving people with VI were published before 2014. The final dataset, therefore, reflects a 10‑year window of published work, which aligns with the relatively recent adoption and development of CBPR approaches within VI research [33].
2. Qualitative research adopting a community‐based participatory approach was our focus. This criterion ensures focus on studies employing collaborative approaches that acknowledge the lived experiences and expertise [7] of people with VI.
3. Studies involving participants with any form of VI were included, thus recognising the highly individualised impact of VI across different individuals [16].
4. Publications in English, Arabic, Greek, Hindi, Italian, Spanish or Urdu were included based on the authors' language proficiency, enhancing the representation of diverse cultures that have developed valuable adaptation strategies.
5. Grey literature that met quality assessment criteria was included, following established systematic review practices to maintain methodological rigour while incorporating valuable non‐academic sources [34].
6. Case studies using CBPR methodology were included to explore context‐appropriate solutions developed through participatory approaches [6].
7. Studies were included regardless of the level of CBPR engagement, encompassing consultation, collaboration and user‐driven approaches. This allowed for a comprehensive mapping of participatory depth, and enabled identification of patterns in engagement levels that is a key consideration given the potential implications of predominantly lower‐level involvement in existing VI research.
8. We included only studies that implemented a CBPR approach, excluding papers that described the CBPR conceptually without reporting an implemented study.

Exclusion Criteria:

1. Studies that only describe CBPR without implementation were excluded, ensuring a focus on research that generates tangible outcomes and user‐centred solutions [6]. This criterion filters out studies that discuss CBPR theoretically, emphasising those that contribute to real‐world impact and practical application.
2. Research involving participants without VI was excluded to ensure that all included studies specifically address the unique challenges of VI, which require specialised research considerations [17]. However, if a study included both VI and non‐VI participants, it was retained in the review, with the analysis focused exclusively on the data pertaining to individuals with VI.

### 2.4 Study Selection and Data Extraction

Two independent reviewers (A.T. and J.K.) screened titles and abstracts, followed by full‐text assessment of potentially eligible studies, following established systematic review practices [35]. A third reviewer (K.P.) resolved any discrepancies. Data extraction was performed using a standardised form based on the Cochrane Handbook recommendations [36]. During full‑text screening, several publications that initially appeared eligible were excluded upon closer examination. In most cases, exclusions occurred because the paper described participatory approaches only conceptually without implementing CBPR methods; involved participants who did not have VI; or used general qualitative or observational designs without meeting the criteria for CBPR. These exclusions resulted from full‑text assessment, where methodological details not evident in titles or abstracts became clear, and also ensured that only studies employing genuine participatory approaches with VI participants were included.

Alongside the procedural and methodological characteristics, we systematically extracted data on CBPR partnership dimensions. These included identifying the stages of the research process at which VI non‑academic partners were involved (e.g., priority setting, research design, data collection, analysis and dissemination), the depth of their engagement (consultation, collaboration and co‑leadership) and the roles they assumed within decision‑making processes. These partnership dimensions were subsequently coded and synthesised alongside methodological and substantive themes to capture not only how CBPR was conducted but also how power‑sharing and co‑production were enacted within each study.

Beyond documenting partnership stages, we extracted data on factors that facilitated or hindered participatory processes, particularly during the initiation and early engagement phases (e.g., recruitment strategies, communication practices, community readiness and researcher–participant alignment). We also extracted information on how trust was built and sustained within partnerships, including the use of shared charters, co‑developed expectations, ongoing dialogue, relational labour and governance structures that supported transparency and power‑sharing. These relational and contextual elements were included to capture the deeper partnership dynamics that shape CBPR implementation.

### 2.5 Quality Assessment

The methodological quality of included studies was assessed using the JBI Critical Appraisal Checklist for Qualitative Research [37]. This tool evaluates 10 criteria, including methodological congruity, researcher positioning, participant representation and ethical considerations. A.T. and J.K., as independent reviewers, conducted the quality assessment, with disagreements resolved through discussion, while further disagreements were resolved through consultation with a third reviewer (K.P.).

All studies meeting the inclusion criteria were retained regardless of their quality appraisal scores. The JBI Checklist was used to assess methodological strengths and limitations, but no studies were excluded based on low methodological quality. This approach aligns with established qualitative synthesis guidance, recognising the methodological diversity typical of CBPR designs.

### 2.6 Data Transparency

PRISMA 2020 guidelines for systematic reviews were adhered to Page et al. [28]. All data are presented in Table 1 and described in the text. This review was originally started in December 2023 and last updated in February 2025. It was preregistered and published via PROSPERO (Registration Number: CRD42024493802 Protocol).

**Table 1:** Summary of included studies.

Sr. No.	Author(s) (year)	Demographics (incl. number of participants, age range, inclusion of blind participant or dual sensory impairment)	Definition, measurement or eligibility of VI.	Methods, and level of engagement	Main findings	Recruitment strategy	Meeting format, frequency and duration	Communication mode	Accessibility notes
1.	Agarwal and Agarwal [38]	13 (particular focus on 4) school children with VI in Bangalore, India. 13–18 years old. Blind participants included. No dual sensory impairment reported.	No particular definition of VI. Eligibility through admission at a specialist blind school.	Ethnography through collaboration in creating tactile art. Collaboration	Insights on how children with VI perceive their world and create realities.	School‐based recruitment of 13 blind students from Belaku Academy in Bangalore via an immersive art programme.	Eighteen‐day fieldwork including three days of full immersion; four clay workshops with selected participants.	Interaction, storytelling, peer review and co‐creation during face‐to‐face group activities.	Tactile materials (tactile exploration, adaptive pacing, abstraction through clay) tailored to students' sensory strengths.
2.	Bao et al. [26]	18 high school students with VI. 14–21 years old. Inclusion of blind participants was not reported. No dual sensory impairment reported.	No particular definition of VI. Eligibility through school identification of VI.	Experimental design with gameplay to improve self‐efficacy. Consultation	Exergames improved task and scheduling self‐efficacy by enhancing emotional engagement, physical activity and social interaction.	Students with VI aged 14–21 were recruited from a specialist school via teacher coordination.	Activities spanned design workshops and a 2‐day experiment, with ~40‐min gameplay sessions.	In‐person one‐on‐one interviews, group discussions and audio‐tactile interaction during gameplay.	Tactile tunics and auditory feedback enabled nonvisual play; design was co‐created with students.
3.	Chiplunkar et al. [39]	84 IT professionals with VI. 21–45 years old. Blind participants included. No dual sensory impairment reported.	No particular definition of VI. Eligibility through VI‐support organisations.	Participatory design with VI users to co‐develop and evaluate an audiohaptic drawing tool for colour‐blindness. Collaboration	Audiohaptic tool enhanced collaborative co‐creation, surpassing traditional notation in accessibility and generative potential.	VI adults (ages 21–45) were recruited via local accessibility councils in Indiana.	Interviews, contextual inquiry and two 1‐h co‐design workshops over 6 weeks.	Online interviews via Zoom, tactile exploration and collaborative prototyping with sighted participants.	Audio‐haptic feedback, tactile UI elements and shared tools supported inclusive collaboration.
4.	Feucht and Holmgren [40]	Four undergraduate students with VI. Age range not reported, mean age 22.5 years. Blind participants included. No dual sensory impairment reported.	Defines VI as legal blindness. Eligibility through registration as blind or partially sighted.	Four‐phased development and research design process for tactile map creation. Involvement	Customisation of local maps for individual users.	Four undergraduates with low vision were purposefully sampled from a Midwestern university.	Four‐phase process including in‐person interviews and field tests; sessions ranged from 30 to 90 min.	Face‐to‐face semi‐structured phone interviews, in‐person site visits and tactile map exploration over a month period.	Maps used braille and microcapsule media; feedback led to personalised layouts, symbols and orientation aids.
5.	Guzman‐Orth et al. [41]	School staff and 17 school students with VI. Age range not reported, includes students from Year 1 to Year 12, corresponding roughly to 5–18 years old. Blind participants included. No dual sensory impairment reported.	No particular definition of VI. Eligibility through disability category of VI in the school records	Participatory design for accessible tests with school community members. Consultation	Creation of an accessible English‐language proficiency test for people with VI.	School‐wide call and teacher referrals; 17 K–12 VI or blind students were purposefully sampled statewide.	Mixed: five in‐person cognitive lab sessions (25–90 min each), plus online consultation and virtual focus activities over 3 months.	Retrospective interviews, observer protocols and assistive‐tech‐supported test interactions via Google Meet and emails.	Tactile graphics, screen readers, Braille devices and individualised accommodations in digital testing reviewed collaboratively.
6.	Higginbottom et al. [42]	32 Somali refugees with VI, community members and service providers. Age range not reported. Blind participants included. No dual sensory impairment reported.	No particular definition of VI. Eligibility through the Horn of Africa Blind Society, which requires registration as blind or partially sighted.	Three‐phased collaborative focused ethnography on health and social care needs of refugees with VI. Consultation	Sociocultural perceptions of blindness and VI, access to services, isolation and insecurity, and mobility were the main themes that emerged.	Somali participants with VI and caregivers were recruited via the Horn of Africa Blind Society and snowball sampling.	Four focus groups and 41 individual interviews were conducted over three phases, each lasting 30–90 min.	In‐person and telephone interviews in Somali or English, supported by bilingual staff and translated materials.	Audio formats, bilingual consent materials and culturally sensitive facilitation.
7.	Ives et al. [43]	Four people with VI in East Harlem, New York, with diabetes. 31–65 years old. Inclusion of blind participants was not reported. No dual sensory impairment reported.	Defines VI as decreased vision. Measured using the Lighthouse Functional Vision Screening Questionnaire.	Qualitative design using photovoice methodology. Consultation	Photovoice was an effective tool in identifying the needs of participants, educating and raising awareness.	Community outreach and local clinics; eligibility based on vision function issues and willingness to engage with photography.	Three photovoice workshops over 1 month, plus individual sessions and ongoing community board involvement.	Face‐to‐face workshops, individual interviews and multimedia storytelling through photography and film.	Cameras were adapted with bump dots and magnifiers; materials used bold fonts and well‐lit displays.
8.	Keene [15]	Four blind young adults and nine high school physical education (PE) teachers. Age range not reported. Blind participants included. No dual sensory impairment reported.	No particular definition of VI. Participants self‐identified as blind.	Fifteen online meetings to construct a tool for PE teachers. Collaboration	Three themes emerged with mixed opinions about practicality of the tool: (1) ‘You're gonna have to get to know the person’: awareness of needs; (2) ‘For a teacher that's on their own…this is phenomenal’: more than a planning tool; (3) ‘I should meet with the student’: conversations for student input.	Four blind youth (ages 18–22) based on recent U.S. high school graduation.	Fifteen Zoom meetings over 19 weeks, split into two stages: needs exploration and co‐construction.	Virtual meetings with collaborative discussion, transcription analysis and iterative feedback loops.	Tool co‐developed with screen reader compatibility.
9.	Pigeon et al. [27]	49 participants in France, including VI adults and children (via parental input), caregivers, professionals and researchers. Age range not reported. Blind participants included. No dual sensory impairment reported.	The definition of VI was aligned with the French disability and public‐health framework. Eligibility through VI‐support organisations.	Focus groups, Delphi method and advisory board consultation regarding a survey. Collaboration	Final survey with 191 questions capturing the diversity of people with VI.	49 participants via consortium contacts and snowball sampling, including blind individuals, caregivers, professionals and researchers.	Four‐phase process with online questionnaires, five focus groups, two Delphi rounds and final consultations over 4 months.	In‐person and virtual (unspecified) focus groups, online surveys, phone calls and email exchanges.	Survey co‐created via iterative feedback loops (e.g., Word versions, breaks).
10.	Schölvinck et al. [6]	Adults with VI; children represented via parents; older and younger adults; and people with varying ophthalmological conditions: 89 consumers, 784 and 631 respondents. Age range not reported. Inclusion of blind participants was not reported. Includes deaf‐blind participants.	No particular definition of VI. Eligibility through registration as visually impaired or deaf‐blind.	Dialogue Model, multi‐phase (interviews, focus groups, surveys, dialogue). From consultation to involvement.	Multiple major themes are highlighted in medical and sociopsychological research agendas.	89 people with VI and related stakeholders via consumer associations and purposeful sampling.	Multiphase interviews, focus groups and surveys over 1 year.	In‐person meetings, paper and online surveys, and follow‐up dialogue sessions with stakeholders.	Dialogue model supported layered engagement.
11.	Tanui [44]	Six young adults with VI, and four school committee members from Elgeyo‐Marakwet and Kisumu, Kenya; mostly braille‐using men with childhood‐onset VI; varied education/employment; plus sighted school representatives. Age range not reported, focuses on young adults. Inclusion of blind participants was not reported. No dual sensory impairment reported.	No particular definition of VI. Identified through CBR or school/community networks.	Grounded theory, case study, with interviews, photovoice and observations. Involvement to collaboration level.	Community‐based rehabilitation helped reduce poverty among young adults with VI and their families. It also enhanced their decision‐making abilities and improved access to resources through support from community stakeholders and agencies.	School committee and community liaison.	Four in‐person interviews and photovoice over 2 months.	Face‐to‐face.	Braille materials and tactile maps used.
12.	Theodorou and Meliones [5]	11 adult participants with VI. 30–60 years old. Blind participants included. No dual sensory impairment reported.	VI participants are defined as blind or with severely impaired vision. Recruited through a VI‐support organisation.	Interviews with people with VI at the first stage of navigation app development. Consultation level	Identifies everyday practices and psychological features of people with VI for use in technology development. Community‐based rehabilitation strategy to promote social change and enable people with VI.	Eleven blind and VI young adults through the Lighthouse for the Blind of Greece.	Single‐session in‐person interviews lasting at least 45 min each, conducted during early app development.	In‐person interviews focused on navigation habits, psychological factors and app feature feedback.	Interviews explored fears, habits and training needs; findings informed a tailored training app framework for app adoption.

### 2.7 Study Selection Process

**Figure 1:**
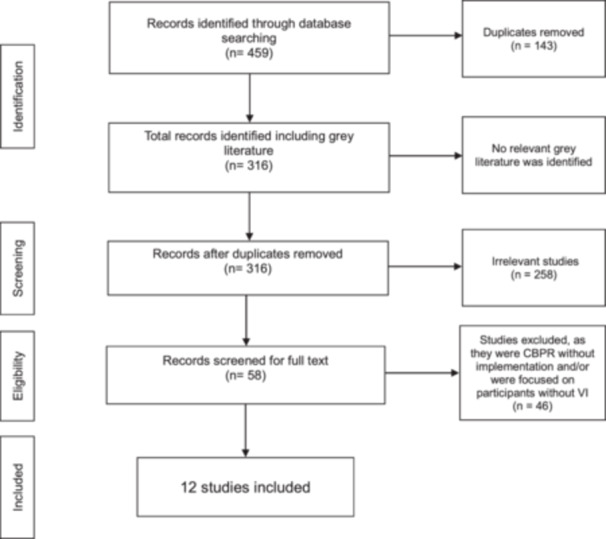
Prisma flowchart to show study selection process.

The PRISMA flow diagram (Figure 1) illustrates the systematic process of study identification, screening and inclusion. Two independent researchers (K.T. and J.K.) conducted parallel searches to ensure accuracy. Both researchers searched for studies, removed duplicates and screened the remaining records by reading titles, abstracts and then full texts. At every step, both researchers followed a clear set of inclusion criteria to make sure only relevant studies were considered. The researchers ultimately identified the same 12 studies that fully met the criteria for inclusion. This systematic approach ensured comprehensive coverage of relevant research while maintaining methodological rigour in accordance with PRISMA guidelines [28, 45].

During full‑text screening, all potentially eligible articles were examined in detail, and specific reasons for exclusion were recorded in accordance with PRISMA 2020 recommendations. Studies were excluded at this stage when they did not employ a CBPR approach in their research, when the methodology was purely observational and qualitative and therefore not aligned with the qualitative focus of this review, or when the study population did not include individuals with VI or did not provide extractable VI‑specific data. Additional exclusions occurred when CBPR was discussed only conceptually without implementation, when the available methodological information was insufficient to determine whether participatory engagement had taken place, or when the record consisted only of a conference abstract or grey literature source that lacked the necessary methodological detail for eligibility.

### 2.8 Characteristics of Included Studies

While our search strategy identified publications rather than study registries, we examined all full texts to determine whether any publications originated from the same underlying study. No such duplication was identified; each included publication reported on a distinct study. The 12 studies included in this systematic review represent diverse methodological approaches and geographical contexts in CBPR involving VI participants. As shown in Table 1, these studies encompass various participation types, group sizes and content themes. The studies were conducted in different continents, including North America (*n* = 5), Europe (*n* = 3), Asia (*n* = 2) and Africa (*n* = 2), providing cross‐cultural perspectives on VI experiences. Publication dates ranged from 2014 to 2024, with half of these studies (*n* = 6) published within the last 5 years, indicating growing interest in participatory approaches with VI populations. The studies varied considerably in sample size, ranging from small focus groups of four participants to larger surveys involving over 200 respondents. All 12 included papers were published in English, despite our broader eligibility criteria allowing for publications in seven languages. This likely reflects the dominance of English‑language publication within CBPR and VI research.

However, not all surveys met the criteria for CBPR; only those in which survey data were used collaboratively (e.g., to inform subsequent co‐designed interventions or guide community‐led decision‐making) were considered eligible. Most studies (*n* = 9) employed multiple participatory methods, reflecting a growing trend toward methodological triangulation in CBPR. These methods included focus groups, which facilitated collective discussion and shared reflection among participants; creative workshops, which used artistic and design‐based activities to elicit experiential knowledge; photovoice, a participatory technique in which individuals use photography to document and communicate aspects of their lived experience; and final consultations, where participants reviewed findings and contributed to shaping recommendations or outputs. For instance, Tanui [44] combined interviews, focus groups and photovoice to explore both community perceptions and personal narratives of young adults with VI in Kenya. Similarly, Guzman‐Orth et al. [41] employed participatory design workshops, iterative consultations with students and teachers, and structured evaluations to co‐develop accommodations for language testing.

By integrating diverse methods, these studies captured both generalisable insights, such as survey data on service needs, and nuanced context‐specific narratives, including individual psychosocial experiences. This triangulation enhanced the richness, validity and cultural relevance of findings, ensuring they reflected the diverse perspectives within the VI community. Collectively, the included studies represent the voices of approximately 580 participants with VI, spanning a wide range of age groups, aetiologies and onset periods and offering comprehensive insights into the lived experiences and priorities of this heterogeneous population.

To better understand the feasibility, accessibility and replicability of CBPR in VI research, we examined how participants were recruited, the format and frequency of meetings, and the communication platforms used. This mapping provides practical insights into the logistical dimensions of inclusive research and highlights contextual factors that shaped participant involvement. Table 1 also summarises these engagement logistics, offering a complementary perspective to the methodological overview in the same table.

### 2.9 Data Synthesis Process

The thematic synthesis incorporated partnership‑related codes, allowing us to examine how, when and to what extent non‑academic VI partners participated across different stages of the research process. Codes relating to facilitators, barriers and trust‑building mechanisms were integrated into the thematic synthesis to examine not only procedural elements of CBPR but also the relational conditions that enabled or constrained meaningful partnership.

More precisely, our synthesis process employed a dual‐coding approach to analyse both the methodological aspects (how) and the content themes (what) addressed in the included studies. Using NVivo software (Version 12, QSR International), we conducted a qualitative thematic synthesis following the six‐phase approach outlined by Braun and Clarke [46]: This involved: (1) familiarisation with the data, (2) generating initial codes, (3) searching for themes, (4) reviewing themes, (5) defining and naming themes and (6) producing the report.

The synthesis process involved systematic coding by two independent coders (AT and JK) as well as theme development following established qualitative synthesis methods [47]. To ensure reliability, 10% of the analysed data was reviewed by an additional researcher (MP), following recommended practices for qualitative research validation [48].

The initial coding framework focused on methodological approaches (‘how’) employed in CBPR studies involving people with VI. This included coding for participatory methods, data collection techniques and levels of participant engagement. Following this, a secondary analysis was conducted to identify the substantive content themes (‘what’) addressed in these studies, including specific issues, challenges and solutions identified through the CBPR process.

## 3 Results

### 3.1 Types of Participation

Our analysis of the methodological approaches employed across the 12 included studies revealed a spectrum of participatory methods, which we grouped into six distinct types. These ranged from minimally participatory methods, where individuals with VI were consulted for data collection, to deeply collaborative approaches involving co‐design, shared decision‐making and iterative feedback. Notably, many studies employed multiple approaches, resulting in cumulative frequencies that exceed the total number of studies.

Final consultation emerged as the most frequently employed method (*n* = 8), typically involving participants with VI offering feedback on pre‐developed interventions or proposed solutions. This stage often functioned as a reflective process, allowing participants to assess both the relevance and accessibility of initiatives. For instance, Tanui [44] used photovoice to foreground the lived experiences of young adults with VI in rural Kenya. Participants revealed a paradox in which services designed to promote inclusion were themselves exclusionary. A community‐based rehabilitation office, intended to serve VI individuals, was described as ‘hidden between offices and inaccessible with no signs or directions’ (p. 95).

Guidance approaches (*n* = 6) were characterised by VI participants actively shaping research priorities and methodologies, positioning themselves as co‐creators rather than informants. This reflects a commitment to inclusive and democratic research practice, where lived experience is central to meaningful design. Guzman‐Orth et al. [41] exemplify this approach through their participatory design framework for accessible English language proficiency assessments. Their cognitive lab study embraced the ethos of ‘Nothing about us without us’ of Charlton [14], emphasising early and sustained involvement of disability and school community members in iterative development cycles. This method extended beyond consultation to collaboration across design, evaluation and refinement stages, demonstrating the transformative potential of participatory research.

Creative discussion methods (*n* = 5) enabled imaginative and embodied engagement, often sparking spontaneous collaboration and playful co‐design. These approaches encouraged VI participants to experiment, express ideas audibly and physically and shape development in real time. In Bao et al. [26], guided workshops and improvised musical challenges allowed students to co‐create game elements through movement, listening and peer interaction. One exchange ‘You sing, we'll follow you. Please slow down… OK, one, two, three…’ [26], p. 238 captured the spirit of collaborative rhythm and emotional resonance. While researchers facilitated the structure and tools, the creative direction and tempo were led by participants, highlighting moments of peer‐led improvisation and shared agency. Auditory and tactile prompts, such as texture‐coded sensors and melodic imitation, facilitated active exploration and peer‐led guidance, transforming abstract design into performative action.

Survey‐based participation (*n* = 4) involved co‐development of questionnaires with VI community members, foregrounding collective priority‐setting and shared authorship. Pigeon et al. [27] exemplify this approach through their national survey project in France, which included focus groups, Delphi consensus rounds and final consultation. The Delphi method [49, 50] involves multiple stages of anonymous feedback designed to help diverse stakeholders reach agreement on shared priorities. The resulting 191‐item survey reflected both individual lived experiences and community‐agreed priorities, ranging from practical (‘How long did it take to get a first diagnosis?’) to reflective (‘Are you satisfied with your daily life?’). This participatory depth positioned the survey not merely as a data collection tool, but as a mechanism of empowerment and representation.

In contrast, ratings approaches (*n* = 3) and socio‐demographic data collection (*n* = 2) represented more limited forms of participation. Ratings positioned participants primarily as evaluators of pre‐developed tools or experiences, offering interpretive agency but lacking dialogic or generative qualities. Feedback was often categorical, with minimal opportunity for elaboration. Socio‐demographic data collection, while essential for contextualising findings, reflected an extractive mode where VI individuals were subjects rather than collaborators. These studies were included only when such methods were embedded within broader qualitative CBPR frameworks; for example, when rating tasks were followed by participant‐led discussions or when demographic surveys informed co‐designed interventions. In this context, they serve a comparative function, illustrating how increased depth of participation correlates with more inclusive and emotionally resonant outcomes. Even minimal participation can be meaningfully enriched through strategies such as transforming rating tasks into dialogic sessions or integrating open‐ended reflections that invite personal narratives and co‐authorship.

### 3.2 Group Size

Group size varied considerably across the reviewed studies, reflecting differences in methodological design, research scope and participatory intent. Participation methods were categorised by the scale of engagement, ranging from small, intimate groupings to broader population‐level involvement. Small‐scale approaches (*n* = 9), such as those by Feucht and Holmgren [40] and Ives et al. [43], included fewer than 10 participants and often employed intensive qualitative methods such as photovoice or tactile mapping. These methods, along with advisory boards, focus groups and targeted consultations, fostered close dialogue and enabled deeper exploration of sensitive, context‐specific issues. These settings often created space for personal testimony and emotional nuance. For example, in Tanui [44], a participant shared, ‘At home, it is difficult to be chosen as a leader in the society. Getting married is a problem but I prayed and God answered my prayer’, illustrating the social and spiritual dimensions of lived experience. Studies such as Feucht and Holmgren [40], Ives et al. [43] and Keene [15] involved fewer than 10 participants, allowing for in‐depth, personalised engagement and iterative design processes.

Mid‐sized studies, including those by Agarwal and Agarwal [38], Guzman‐Orth et al. [41] and Pigeon et al. [27], typically involved between 13 and 49 participants. These designs balanced individual depth with broader representation, enabling researchers to capture diverse perspectives while maintaining manageable group dynamics. Many of these studies also employed participatory design frameworks, allowing for meaningful collaboration across stakeholder groups.

In contrast, large‐scale approaches (*n* = 5), such as Schölvinck et al. [6] that engaged over 600 respondents, relied on tools such as surveys and ratings to reach wider participant pools. These methods prioritised representational breadth, capturing data across diverse geographical and demographic categories. While they supported broader generalisation, they offered less opportunity for nuanced exploration or personalised engagement. Schölvinck et al. [6] and Pigeon et al. [27] exemplify this approach, involving hundreds of respondents to inform national‐level agendas and policy frameworks. Additionally, several studies incorporated mixed stakeholder groups, including people with VI, caregivers, professionals and community members, as seen in Higginbottom et al. [42] and Tanui [44]; thereby enriching the diversity of perspectives and enhancing contextual relevance.

While each study size offers distinct advantages, from deep personal insight to broad representational coverage, their capacity to meet participatory standards varies considerably. Small‐scale designs often enable iterative dialogue and co‐construction, aligning closely with CBPR principles [11, 23]. In contrast, large‐scale surveys, though valuable for agenda‐setting, may lack the feedback loops and shared decision‐making that define genuine participation [12]. Without mechanisms for respondent input beyond data collection, such studies risk reinforcing extractive models [14]. Moreover, certain topics such as psychosocial adaptation or cultural navigation may demand intimate formats to surface lived nuance [16], while policy‐oriented questions may benefit from broader sampling [51]. Future CBPR efforts should critically match study size to topic complexity and ensure that participatory claims are substantiated by reciprocal engagement.

### 3.3 Modes of Engagement and Study Logistics

The mapping of engagement logistics revealed considerable variation in how CBPR was enacted across studies. While some projects involved sustained collaboration over multiple sessions [6, 41], others relied on one‐off consultations [5, 26]. Online platforms such as Zoom and Google Docs [15] facilitated flexible participation, though accessibility of digital tools was not consistently reported. Recruitment strategies ranged from school‐based outreach [38] to community liaison [42, 44], with varying degrees of transparency regarding inclusion criteria and participant support.

## 4 Discussion

### 4.1 Content Themes

Within the reviewed methodological approaches, three primary content themes emerged: support services, social experience and research and design innovation. These themes reflect the dual emphasis on systemic provision and lived engagement, as well as the evolving role of participatory research in shaping inclusive tools and agendas.

The support services theme encompassed studies focused on vision screening (*n* = 4), educational support (*n* = 5) and common law policies (*n* = 2). These interventions often addressed structural barriers and unmet needs within formal systems. For instance, [41] documented a shift in educational provision, where a participant noted, ‘There has never been a practice Braille test until now. The opportunity is better now’, reflecting tangible improvements in accessibility. Similarly, Tanui [44] highlighted how community‐based rehabilitation initiatives helped reduce poverty among young adults with VI and their families, while enhancing decision‐making and access to resources.

The social experience theme (*n* = 5) foregrounded leisure, sports and cultural participation as essential dimensions of everyday inclusion. These studies captured personal preferences and the rhythms of daily life. In Bao et al. [26], a student remarked, ‘I would definitely engage in this game… I am busy with school subjects, I'm not certain about twice a week, but I am sure once a week is good for me’, illustrating how participatory design can flexibly respond to individual capacities and aspirations. Such studies emphasised emotional resonance, peer interaction and the importance of designing activities that reflect the lived realities of VI individuals.

Expanding beyond service provision and social engagement, a third thematic strand focused on technology and design innovation. A significant number of studies addressed the development of accessible tools and platforms, including tactile art [38], audiohaptic drawing tools [39], navigation apps [5] and educational planning tools for physical education teachers [15]. These studies often employed participatory design frameworks, enabling VI individuals to shape the functionality, aesthetics and usability of emerging technologies.

Themes related to education and empowerment were also prominent, with studies such as Bao et al. [26], Guzman‐Orth et al. [41] and Tanui [44] exploring self‐efficacy, inclusive testing and community‐driven rehabilitation. These interventions not only addressed immediate educational needs but also fostered long‐term agency and confidence among participants.

Finally, broader policy and research agendas were addressed in studies like Schölvinck et al. [6] and Pigeon et al. [27], which aimed to capture the diverse experiences of people with VI and inform future research and practice. These large‐scale, multi‐phase projects employed tools such as surveys, interviews and consensus‐building methods to identify priorities across medical, social and psychological domains. By centring the voices of VI individuals, these studies contributed to more representative and responsive research frameworks.

Together, these thematic strands underscore the richness and complexity of participatory research with VI communities. They highlight how inclusive methodologies can illuminate both systemic challenges and personal aspirations, ultimately shaping interventions that are more equitable, relevant and emotionally attuned.

### 4.2 Content Domains

While the preceding thematic synthesis highlights the breadth of concerns addressed in studies involving VI participants, it is essential to clarify how these themes specifically reflect the contributions of CBPR. Unlike conventional methodologies, CBPR foregrounds the active involvement of participants in shaping research questions, interpreting findings and co‐designing interventions. This participatory lens not only enriched the thematic scope by bringing emotional nuance, contextual specificity and practical relevance, but also surfaced priorities and insights that may be overlooked in more extractive or top‐down approaches. For instance, the emphasis on leisure preferences, tactile engagement and self‐advocacy emerged through iterative dialogue and co‐analysis, rather than predefined metrics. To further unpack this distinction, the following section maps the substantive content domains addressed across the studies, illustrating how CBPR methodologies enabled deeper exploration of lived experience, systemic navigation and inclusive design.

### 4.3 Accessibility and Technology

Accessibility and technology emerged as a central domain across the reviewed studies, encompassing innovations in assistive devices, digital access and sensory representation. A key subtheme involved abstract object representation, where tactile exploration and mental mapping enabled participants to engage with concepts beyond literal replication. These findings highlighted the cognitive sophistication involved in non‐visual spatial understanding and the creative strategies individuals with VI use to interpret and navigate their environments.

Crucially, CBPR approaches shaped not only the focus of these studies but also the development of the tools themselves. Rather than treating participants as test subjects, researchers engaged them as co‐designers, collaborating on the creation of accessible questionnaires, navigation tools and learning platforms. This co‐creation was achieved through a range of participatory formats in Guzman‐Orth et al. [41], where students and teachers with VI participated in iterative design workshops held in person over several weeks, where feedback was gathered through tactile prototyping, guided discussion and structured evaluation sessions. In Pigeon et al. [27], survey items were developed through a multi‐phase process involving advisory board meetings, Delphi consensus rounds and final consultations. Participants contributed both individually and in group settings, some via online platforms, others through in‐person focus groups, sharing lived experiences and reviewing draft items to ensure cultural relevance and emotional resonance.

These contributions underscore how participatory methods can surface priorities and usability criteria that may be overlooked in more conventional research. By embedding engagement throughout the design cycle, from initial ideation to final validation, these studies produced technological solutions that were not only functional but also empowering, context‐sensitive and emotionally attuned to the needs of the VI community.

Technology‐focused CBPR studies highlight the importance of participatory scale in surfacing usability challenges and cultural misalignments. Tools like those in Chiplunkar et al. [39] and Guzman‐Orth et al. [41] were refined through iterative, small‐group engagement, allowing users to shape design logic and interface accessibility. In contrast, survey‐based evaluations may flag general dissatisfaction but lack the granularity needed to inform redesign. For inclusive technology development, participatory formats must allow for sustained dialogue, testing and adaptation; conditions rarely met in large‐scale studies without embedded feedback loops.

### 4.4 Sensory and Cognitive Processing

This domain explored how individuals with VI perceive, interpret and navigate their environments through non‐visual modalities. Studies [5, 26, 40] highlighted the role of auditory, tactile and olfactory cues in environmental mapping and spatial cognition, revealing sophisticated strategies for constructing mental representations of space. These insights challenge assumptions about sensory compensation and underscore the creative, embodied knowledge that participants bring to everyday navigation.

What distinguishes these findings within a CBPR context is the way participants actively shaped the focus and framing of sensory experience. Rather than being observed through predefined tasks, individuals with VI contributed their own interpretations of how they process information, revealing nuances often overlooked in traditional research. For example, participants described how they associate specific sounds or smells with locations [5], or how they build spatial understanding through partial tactile input [40], insights that informed the design of more intuitive tools and environments [26].

CBPR also illuminated cognitive processing challenges such as forgetfulness, error correction and information overload, particularly in educational contexts. These moments of uncertainty were not treated as deficits but as opportunities for co‐developing adaptive strategies. By voicing their experiences in real time, participants helped researchers identify design features, such as repetition, pacing or multimodal prompts, that could better support learning and memory. In this way, participatory methods not only deepened understanding of sensory and cognitive variation but also positioned individuals with VI as co‐creators of more responsive and inclusive systems.

The complexity of sensory and cognitive adaptation, particularly in relation to onset age, perceptual strategies and neural reorganisation, demands participatory formats that allow for iterative meaning‐making. Studies exploring these dimensions, such as Tanui [44], benefited from small‐group or case study designs that enabled participants to articulate embodied experiences and co‐interpret their significance. Large‐scale surveys may identify prevalence or general trends in adaptation, but they are ill‐suited to capture the layered, often tacit knowledge that emerges through dialogue and reflection. For CBPR to meaningfully engage with sensory and cognitive experience, it must prioritise depth, co‐analysis and the co‐construction of explanatory frameworks [21].

### 4.5 Independence and Daily Living

This domain encompassed everyday functioning and autonomous living, highlighting how individuals with VI navigate mobility, physical activity, personal care and safety. Mobility and navigation emerged as core concerns, often shaped by unpredictable environmental barriers and the need for adaptive strategies [44]. Physical activity was framed not only as a health‐promoting behaviour but also as a source of joy, agency and social connection, particularly when co‐designed to reflect participants' interests and capacities [26].

CBPR approaches were instrumental in surfacing these dimensions. Rather than focusing solely on functional limitations, participatory methods enabled individuals with VI to articulate what independence meant to them, whether through co‐developing inclusive games [26], reflecting on assistive technology use [41] or identifying relational aspects of care [15]. These insights were not imposed by researchers but emerged through iterative dialogue and participant‐led framing.

Safety concerns were also frequently voiced, particularly in relation to public space navigation and street crossing [43]. CBPR allowed these concerns to be contextualised within broader narratives of vigilance, trust and environmental unpredictability; elements often flattened in conventional assessments. This domain underscores how participatory research can reveal the embodied strategies individuals with VI use to navigate risk, and how co‐designed interventions can foster not only physical safety but also psychological confidence and spatial autonomy.

These findings also raise important questions about the relationship between study size and the depth of contextualisation achievable through CBPR. The nuanced insights into mobility, emotional agency and safety, such as those voiced in Tanui [44] and Ives et al. [43], were largely surfaced through small to mid‐sized studies that enabled iterative dialogue and relational framing. In contrast, large‐scale surveys, while useful for identifying broad trends in service access or unmet needs, may struggle to capture the layered, embodied strategies individuals use to navigate daily life. Without mechanisms for feedback and co‐interpretation, such formats risk flattening complexity into generalisable metrics. This domain exemplifies how certain topics, particularly those involving psychosocial nuance and environmental interaction, may require participatory designs that privilege depth over breadth, ensuring that independence is not merely measured but meaningfully understood [13, 14].

### 4.6 Psychosocial Experience

The psychosocial dimensions of VI were shaped by deeply embedded socio‐cultural, familial and religious factors. Cultural perceptions and linguistic stigma often reinforced exclusion, with participants in Tanui [44] describing derogatory terms used for blind individuals in local languages, terms that perpetuated shame and social marginalisation. Family members frequently provided essential support, yet economic constraints and social expectations limited autonomy, particularly when mobility required accompaniment or financial resources.

Religion emerged as both a source of strength and a site of tension. While some participants drew resilience from faith, others encountered barriers to leadership and marriage roles due to prevailing beliefs about disability [44]. Internalised stigma further impacted confidence and participation, with individuals expressing fear of community judgement and a persistent sense of self‐consciousness.

CBPR approaches were critical in surfacing these layered experiences. By creating space for participants to articulate their emotional landscapes and cultural contexts, researchers were able to move beyond surface‐level accounts of psychosocial wellbeing. Rather than framing stigma and exclusion as abstract phenomena, participatory methods revealed how these forces operate in daily life by shaping identity, relationships and aspirations. This depth of insight, grounded in lived experience, underscores the value of CBPR in capturing the emotional and cultural realities that more extractive methodologies might overlook.

Psychological experiences, such as isolation, resilience or identity negotiation, are deeply personal and context dependent. Studies that surfaced these themes most effectively, including Keene [15] and Ives et al. [43], relied on small‐group or longitudinal engagement that fostered trust and emotional safety. In contrast, large‐scale studies may quantify distress but lack the relational scaffolding needed to explore its roots or co‐develop coping strategies. Participatory research in this domain must therefore be designed not only to collect data, but to hold space for vulnerability, reflection and shared meaning‐making; conditions best supported by smaller, dialogic formats [3].

### 4.7 Educational Experience

Educational access and inclusion were explored through advocacy, accommodation and awareness initiatives, with a strong emphasis on challenging exclusionary norms and promoting equitable learning environments. Studies highlighted inclusive leadership practices within community‐based rehabilitation programmes, where young people with VI were actively involved in organisational decision‐making and rights advocacy [44]. These efforts reflected a broader commitment to break the cycle of marginalisation through targeted education and systemic change [43].

Assessment adaptation emerged as a key area of growth, particularly in relation to equitable testing opportunities. Guzman‐Orth et al. [41] documented the introduction of practice Braille tests, co‐developed with students, which marked a shift toward more inclusive and responsive educational provision. These interventions were not simply technical adjustments but reflected a deeper participatory ethos, where learners with VI contributed to the design and evaluation of tools that directly impacted their academic experience.

CBPR approaches were central to these developments. By involving participants in the identification of barriers, the co‐creation of solutions and the evaluation of outcomes, researchers were able to move beyond accommodation toward transformation. The studies demonstrated how participatory methods can foster self‐efficacy, amplify student voice and embed inclusion within both pedagogical practice and institutional culture.

The educational domain further illustrates how study size influences participatory depth. Co‐designed tools and teacher‐student dialogues, as seen in Keene [15], emerged from small‐group formats that allowed for iterative feedback and relational trust. Larger studies may identify systemic barriers or policy gaps, but without sustained engagement, they risk overlooking the interpersonal and contextual factors that shape learning experiences. For CBPR in education to be transformative, it must enable reciprocal learning and co‐construction; features best supported by mid‐sized or small‐scale designs.

### 4.8 Support Systems and Services

Support systems were assessed in terms of their responsiveness, accessibility and relevance to participants' lived needs. Engagement with targeted interventions was generally positive, particularly when programmes offered flexibility and aligned with personal schedules and interests [26]. These findings underscore the importance of tailoring services to individual rhythms rather than imposing rigid structures, an insight that emerged directly from participant feedback.

Healthcare navigation, however, remained complex. Participants described compounding challenges related to managing health conditions alongside VI, with everyday tasks often becoming a source of stress and struggle [43]. These accounts highlighted gaps in service coordination and the emotional toll of navigating fragmented systems.

CBPR approaches played a critical role in surfacing these nuances. By involving individuals with VI in the design, evaluation and refinement of support services, researchers were able to identify barriers that are often overlooked. These included the emotional labour involved in scheduling, such as coordinating transport, negotiating assistance and repeatedly explaining access needs to unfamiliar staff. Another key insight was the importance of relational continuity, where participants expressed a preference for consistent interactions with trusted professionals rather than fragmented or rotating support. These findings highlight how co‐created interventions can reflect not only practical logistics but also the emotional and relational dimensions of daily life.

The richness of informal support systems, whether familial, peer‐based or community‐driven, often lies in their subtlety and cultural specificity. Studies like Higginbottom et al. [42] and Tanui [44] captured these dynamics through mid‐sized, multi‐stakeholder formats that enabled cross‐perspective dialogue. Larger studies may map the presence or absence of support, but without participatory mechanisms for co‐analysis, they risk overlooking how support is experienced, negotiated or withheld. To understand and strengthen these systems, CBPR must enable participants to define what support means in their own terms and to shape the questions being asked [18].

### 4.9 Environmental Interaction

Interactions with physical environments revealed persistent barriers to safe and autonomous navigation for individuals with VI. Poorly adapted public and private spaces impeded mobility, with participants describing uneven terrain, obstructive features and unpredictable hazards [44]. Transport systems also lacked consistent notification or accommodation, leading to repeated disorientation and heightened vulnerability during travel [42].

Despite these challenges, participants demonstrated resourcefulness and resilience through adaptive strategies such as route memorisation, sensory cue integration and spatial mapping [5]. These approaches reflected not only practical ingenuity but also a deep embodied knowledge of space and movement.

CBPR methodologies were instrumental in capturing these insights. By engaging participants in the identification of environmental barriers and the co‐development of adaptive solutions, researchers were able to move beyond observational accounts toward more grounded, context‐sensitive interventions. The participatory lens also revealed emotional dimensions of environmental interaction, such as trust, anxiety and confidence, that are often absent from conventional accessibility audits [52]. These audits typically focus on physical infrastructure and compliance checklists, such as curb heights, signage or tactile paving, without capturing how individuals with VI feel when navigating those spaces or whether they experience safety, autonomy or emotional ease. In doing so, CBPR helped reframe environmental design as a relational and experiential concern, shaped by the voices and strategies of those navigating it daily.

Environmental barriers, whether physical, social or cultural, are often context‐specific and emotionally charged. Studies that illuminated these dimensions, such as Guzman‐Orth et al. [41], did so through participatory methods that allowed for situated storytelling and iterative sense‐making. Large‐scale surveys may identify general patterns of inaccessibility, but they rarely capture how individuals navigate, resist or reframe these barriers in daily life. For CBPR to inform inclusive environmental design, it must centre lived experience and enable participants to shape both the problem definition and the proposed solutions [17, 26].

### 4.10 Relationship Between Methodological Approaches and Content Domains

Analysis revealed meaningful patterns in how different participatory methods shaped the exploration of specific content domains. Small‐scale, dialogic approaches such as advisory boards and focus groups were particularly effective in surfacing psychosocial experiences and support system needs. These methods created space for participants to share emotionally nuanced reflections, such as the shame and visibility associated with using a white cane [5], or the relational and financial constraints of accessing care [44]. The intimacy of these settings enabled trust‐building and deeper engagement with topics that might remain hidden in more structured formats.

In contrast, large‐scale participatory methods such as surveys and rating tools proved valuable for capturing accessibility and technology needs across broader populations. These approaches facilitated comparative insights into assistive device preferences, digital navigation strategies and systemic gaps in service provision [27, 41]. While less dialogic, these methods still reflected CBPR principles when participants were involved in shaping the questions and interpreting the results.

Creative and sensory‐based methods, including tactile modelling and guided observation, were particularly effective in exploring sensory and cognitive processing. In Tanui [44], for example, participants used clay to express abstract thinking and improvisation, revealing cognitive strategies that might not emerge through verbal discussion alone. Similarly, final consultation approaches, often used to validate findings or co‐develop recommendations, frequently addressed environmental interaction challenges, such as inaccessible pavements or unsafe crossings [42].

Studies that combined multiple participatory methods tended to engage a broader range of content domains and yielded more layered insights. This methodological diversity allowed for both breadth and depth, capturing systemic patterns while remaining attuned to individual experience. For example, Guzman‐Orth et al. [41] employed a combination of co‐design workshops, tactile prototyping and iterative feedback sessions with students and educators with VI. This approach enabled participants to shape both the conceptual framing and the practical features of an educational tool, revealing not only usability concerns but also emotional and relational dynamics such as trust in peer‐led learning and the role of rhythm in spatial orientation. Compared to studies that relied solely on surveys or focus groups, this multi‐method design surfaced intersecting priorities across accessibility, pedagogy and identity. As the authors affirmed, ‘Nothing about us without us’ must be enacted not only in principle but through early and sustained opportunities for co‐design and shared decision‐making.

These findings underscore the importance of methodological intentionality in CBPR with VI communities. Our analysis showed that studies using tactile co‐design, iterative feedback or multi‐phase engagement were more likely to illuminate emotional, relational and spatial dimensions of lived experience, such as trust in navigation, anxiety around scheduling or the role of rhythm in learning. In contrast, studies relying on single‐method approaches, like standalone surveys or rating tasks, tended to surface narrower priorities and missed opportunities for deeper co‐authorship. The choice of engagement approach shaped not only what was explored, but also how participants defined relevance and envisioned change. However, several critical gaps remain, including limited reporting on how co‐creation was facilitated, inconsistent attention to emotional labour and underrepresentation of younger participants and intersectional identities. These gaps warrant focused attention in future research.

While CBPR offers substantial benefits for generating context‑rich, culturally grounded insights, it also presents several challenges that shape its practical implementation in VI research. Participatory approaches are often time‑ and resource‑intensive, requiring prolonged engagement, iterative feedback and sustained relationship‑building; demands that may exceed the capacity of conventional project timelines or funding structures [8, 13]. Even within participatory frameworks, power imbalances can persist: researchers frequently retain control over agenda‑setting, methodological decisions and institutional processes such as ethics governance, which may inadvertently limit opportunities for genuine co‑production [12, 14]. Institutional and structural constraints can further restrict flexibility, particularly when risk‑averse or procedurally rigid environments limit the ability to adapt methods in response to community priorities [7]. Additionally, CBPR carries the risk of tokenism when involvement is limited to late‑stage consultation or when participatory activities are symbolically included without mechanisms for shared authority or decision‑making [32, 51]. Acknowledging these limitations underscores that CBPR, while powerful, requires intentional design, equitable power‑sharing practices and supportive institutional conditions to avoid reproducing the exclusions it aims to challenge and to fully realise its transformative potential.

### 4.11 Limitations and Recommendations

Although our search strategy spanned 1993–2024, all included studies were published after 2014. This does not reflect a limitation of the search but rather highlights the recent emergence of CBPR in the field of VI. Participatory approaches appear to have gained traction only within the past decade, which may explain the absence of earlier publications despite the wider search window.

While the studies reviewed demonstrate methodological creativity and cultural breadth, their limited number raises important concerns about the broader landscape of VI research. Despite the methodological richness and cultural diversity of the 12 included studies, the overall volume of CBPR research in VI remains disproportionately low. This scarcity is particularly concerning given the well‐documented disconnect between clinical innovation and lived experience in VI contexts. The underrepresentation of CBPR suggests systemic barriers such as limited funding for participatory methods, institutional inertia favouring traditional designs and a lack of training in inclusive research practices, that continue to constrain meaningful engagement. While some studies demonstrated promising outcomes through collaboration and co‐design, the predominance of consultation‐level engagement reflects a broader trend of tokenism rather than transformation. If CBPR is to fulfil its potential as a vehicle for equity and innovation, future research must move beyond rhetorical endorsement and invest in sustained, community‐led partnerships that challenge conventional hierarchies and centre lived expertise from the outset.

Additionally, the relatively small number of studies meeting the inclusion criteria is noteworthy, particularly given the substantial volume of technology‐related research in the field of VI. However, this outcome appears to reflect the current methodological landscape rather than limitations in the search strategy. Many technology‐focused studies reference participatory principles or involve user feedback, but do not implement CBPR as defined by shared decision‐making, collaborative design or iterative partnership; such studies were therefore excluded. In addition, we required studies to report on implemented CBPR processes rather than conceptual descriptions, which further reduced the eligible pool. Together, these factors suggest that fully realised CBPR remains underutilised in VI research, particularly within technology development, despite increasing interest in user involvement.

Second, there is also a notable scarcity of studies employing full co‐researcher partnerships, where VI individuals participate as equal collaborators throughout the research process. This absence is particularly striking given that studies with higher levels of participation often yielded richer, more systemic insights. For example, Guzman‐Orth et al. [41] involved VI students and educators in co‐design workshops, tactile prototyping and iterative feedback sessions, which surfaced not only usability concerns but also emotional, pedagogical and relational dimensions such as trust in peer‐led learning and the role of rhythm in spatial orientation. In contrast, studies relying solely on surveys or rating tasks tended to capture narrower priorities, often limited to predefined categories or individual preferences. Future CBPR should prioritise genuine power‐sharing approaches that move beyond consultation toward co‐production, enabling participants to shape research questions, methods and interpretations from the outset.

Third, there is limited evidence of systematic translation of CBPR findings into sustainable interventions or policy change. While many studies documented participant needs and aspirations, few demonstrated clear pathways for implementation. For instance, Ives et al. [43] used photovoice to surface environmental and health‐related barriers for people with VI and diabetes, yet the study focused primarily on awareness‐raising rather than long‐term service redesign or policy uptake. Similarly, Guzman‐Orth et al. [41] co‐developed accessible assessments, but did not report follow‐through into institutional adoption or curriculum reform. This represents a missed opportunity to leverage the transformative potential of participatory research.

On top of the above, technological innovation in CBPR methodologies remains underexplored. Despite the promise of digital tools to enhance accessibility and reach, few studies employed technology‐mediated approaches specifically adapted for VI participants. Chiplunkar et al. [39] stand out for their co‐development of an audiohaptic drawing tool, which enabled collaborative creation and surpassed traditional notation in accessibility. However, most other studies relied on in‐person formats, with limited use of remote platforms or adaptive digital interfaces. Developing and evaluating such tools could expand participation, particularly for geographically dispersed or multiply‐marginalised individuals, namely those experiencing overlapping forms of exclusion, such as due to disability, race, gender or socioeconomic status.

Notably, several studies lacked detailed reporting on meeting frequency, duration and communication modes; a limitation that constrains replication and evaluation of participatory depth.

Another limitation is that none of the included studies specifically recruited or analysed individuals with dual sensory impairment (DSI), despite evidence that the co‑occurrence of vision and hearing loss is relatively common and associated with poorer psychosocial, participation and health outcomes compared with single impairments [53, 54]. The absence of DSI representation limits the transferability of our findings to this group, who may face distinct accessibility and engagement challenges within participatory research.

A separate limitation relates to the limited reflexive reporting within the included CBPR studies. Very few studies explicitly described how researchers' positionalities, backgrounds or institutional roles influenced key stages of the participatory process, such as how research priorities were negotiated, how power imbalances were managed when disagreements arose, or how decisions regarding design, analysis or dissemination were shared with community partners. Likewise, mechanisms commonly used in CBPR to support trust and transparency (e.g., community advisory boards with decision‑making authority, co‑authorship agreements, reflexive field notes or structured debriefing sessions) were rarely documented. This gap makes it difficult to assess how relational dynamics, researcher assumptions or institutional constraints may have shaped participation quality or knowledge co‑production across the studies.

On the same note, we acknowledge that reflexivity was relevant to our own review process. As researchers working with VI people and using participatory designs, we continually reflected on how our professional identities, and our different perspectives shaped by backgrounds in psychology, neuroscience, multisensory integration and assistive technologies, as well as education; prior or current experience with VI communities; assumptions about what constitutes a meaningful participation; and familiarity with CBPR frameworks, could influence our interpretation of engagement depth and partnership quality. These reflexive considerations informed our coding and synthesis; however, we recognise that our positionalities inevitably shaped how we interpreted the relational aspects of CBPR within this review.

Finally, intersectionality remains insufficiently addressed. Most studies treated VI participants as a homogeneous group, with limited attention to how age, gender, socioeconomic status, additional disabilities or cultural background shape experience. Higginbottom et al. [42] is a notable exception, exploring the sociocultural perceptions of blindness among Somali refugees and highlighting how migration, ethnicity and systemic exclusion intersect with VI. Future CBPR must explicitly engage with these layered identities to ensure that resulting interventions are inclusive, equitable and contextually grounded.

## 5 Conclusion

This review is a first attempt to systematically synthesise qualitative CBPR studies involving individuals with VI. Drawing on 12 peer‐reviewed studies published between 2014 and 2024, we conducted a thematic synthesis focused on participatory depth, methodological diversity and lived experience. Each study was appraised for its level of engagement, demographic context and contribution to seven key domains: accessibility and technology, sensory and cognitive processing, independence and daily living, psychosocial experience, educational experience, support systems and services and environmental interaction.

From accessibility and sensory processing to psychosocial experience and environmental interaction, the studies reveal how participatory approaches can surface nuanced, context‐rich insights that might otherwise remain obscured. Crucially, these domains were not explored in isolation but were shaped through collaborative methodologies that centred lived experience, relational knowledge and co‐constructed meaning.

Across the aforementioned seven domains, CBPR enabled participants to articulate priorities, challenge assumptions and co‐design tools and interventions that reflected their realities. Whether through tactile modelling, co‐created questionnaires or iterative design workshops, the participatory ethos fostered trust, relevance and emotional resonance. Importantly, methodological diversity, ranging from small‐group dialogue to large‐scale surveys, was not merely a technical choice but a strategic means of amplifying different facets of the VI experience. Studies that embraced multiple methods tended to yield more holistic understandings, reinforcing the value of flexibility and responsiveness in participatory design.

However, not all domains benefited equally from this methodological diversity. Psychological experience, sensory and cognitive adaptation and support systems were often explored through small‐scale formats without complementary broader engagement, limiting opportunities for triangulation or policy translation. Conversely, domains such as environmental interaction and educational experience were sometimes addressed through large‐scale surveys without iterative feedback loops, raising questions about the depth of participation. These imbalances suggest that certain areas remain methodologically siloed, and that future CBPR efforts should intentionally match study design to topic complexity, ensuring that each domain is explored through both intimate, dialogic methods and scalable, inclusive tools.

At the same time, this review identified critical gaps that must be addressed to advance the field. The limited use of full co‐researcher partnerships, the underutilisation of technology to enhance participation, the lack of implementation pathways and the insufficient attention to intersectionality all point to areas where CBPR practice must evolve. Addressing these gaps is not only a matter of methodological rigour but of justice, ensuring that participatory research lives up to its transformative promise.

In addition to thematic and methodological insights, this review also foregrounds the practical dimensions of CBPR implementation. By mapping recruitment strategies, meeting formats and communication modes, we highlight the often‐overlooked logistics that shape the depth and sustainability of participation. While some studies demonstrated thoughtful, iterative engagement, others lacked transparency around duration, frequency or accessibility adaptations. These omissions limit replicability and obscure the relational labour underpinning participatory work. Future CBPR studies in VI should prioritise clear documentation of engagement processes, including logistical choices and accessibility adaptations, to strengthen methodological transparency and support equitable research design.

## Author Contributions

**Aikaterini Tavoulari:** conceptualization, investigation, methodology, project administration, resources, data curation, software, formal analysis, visualization, writing – original draft, writing – review & editing. **Jibraan Kidwai:** investigation, methodology, formal analysis, data curation, visualization, software, writing – original draft, writing – review & editing. **Michael Proulx:** funding acquisition, conceptualization, investigation, supervision, methodology, software, formal analysis, data curation, resources, writing – review & editing. **Karin Petrini:** funding acquisition, conceptualization, investigation, supervision, resources, data curation, software, formal analysis, project administration, methodology, visualization, writing – review & editing.

## Ethics Statement

The authors have nothing to report.

## Conflicts of Interest

The authors declare no conflicts of interest.

## Data Availability

The data that support the findings of this study are available from the corresponding author upon reasonable request.
